# Field Study on the Microclimate of Public Spaces in Traditional Residential Areas in a Severe Cold Region of China

**DOI:** 10.3390/ijerph16162986

**Published:** 2019-08-20

**Authors:** Yujie Lin, Yumeng Jin, Hong Jin

**Affiliations:** School of Architecture, Harbin Institute of Technology, Key Laboratory of Cold Region Urban and Rural Human Settlement Environment Science and Technology, Ministry of Industry and Information Technology, Harbin 150006, China

**Keywords:** sever cold region, traditional residential area, street, courtyard, microclimate, field measurements

## Abstract

As residential environment science advances, the environmental quality of outdoor microclimates has aroused increasing attention of scholars majoring in urban climate and built environments. Taking the microclimate of a traditional residential area in a severe cold city as the study object, this study explored the influence of spatial geometry factors on the microclimate of streets and courtyards by field measurements, then compared the differences in microclimate of distinct public spaces. The results are as follows. (1) The temperature of a NE-SW (Northeast-Southwest) oriented street was higher than that of a NW-SE (Northwest-Southeast) oriented street in both summer and winter, with an average temperature difference of 0.7–1.4 °C. The wind speeds in the latter street were slower, and the difference in average wind speed was 0.2 m/s. (2) In the street with a higher green coverage ratio, the temperature was much lower, a difference that was more obvious in summer. The difference in mean temperature was up to 1.2 °C. The difference in wind speed between the two streets was not obvious in winter, whereas the wind speed in summer was significantly lower for the street with a higher green coverage ratio, and the difference in average wind speed was 0.7 m/s. (3) The courtyards with higher SVF (sky view factor) had higher wind speeds in winter and summer, and the courtyards with larger SVF values had higher temperatures in summer, with an average temperature difference of 0.4 °C. (4) When the spaces had the same SVF values and green coverage ratios, the temperature of the street and courtyard were very similar, in both winter and summer. The wind speed of the street was significantly higher than the courtyard in summer, and the wind speed difference was 0.4 m/s.

## 1. Introduction

As climate problems have become progressively severe in recent years, more attention has been given to urban microclimate environments. Previous studies have demonstrated the impact of urban microclimates, to various extents, on the comfort of residents during outdoor activities, the spread of city pollutants, and the energy consumption of buildings, as well as other aspects [1,2,3,4,5]. Many factors affect microclimates, including urban climate, natural topography, green space systems, river systems, and urban morphology [6,7]. Among these factors, urban morphology is tightly tied to the architectural environment. This is represented as urban geometry at the macro level, urban textures at the middle level, and at the micro level it is divided into the layout and geometry of buildings, streets, and squares. Street spaces connect various building groups in tandem, serving as the necessary space by which residents in residential areas commute daily. Acting as a buffer space outside buildings, courtyards provide a milder microclimate to a certain extent, giving residents an open and natural space where they can engage in outdoor communication and activities.

In terms of the urban street microclimate, studies have shown that street aspect ratios, green coverage ratios, and land surface materials wield a significant impact on the physical environment of a street space [8,9,10,11,12,13]. Among various elements, Mohajeri found that the orientation of a street imposes the greatest influence on the amount of solar radiation that the street space can obtain [14]. Chatzidimitriou’s study pointed out that, in winter, NE-SW oriented streets have a higher temperature, and the difference in wind speed between streets with different orientations at the same moment can reach up to 2.7 m/s [15]. According to research by Sözen, the mean wind speed of N-S (North-South) oriented streets is 3.7 m/s faster than that of E-W (East-West) oriented streets and 1.1 m/s higher than that of NE-SW oriented streets [16]. Comprehensively considering the thermal comfort and solar energy available in winter, Ali-Toudert pointed out that NE-SW and NW-SE oriented streets are the best choices [17,18]. In regards to street greening, Lee found that an increase in green coverage ratio is conducive to reducing temperature, with a cooling effect that is more significant during the daytime than nighttime [19]. Moreover, Srivanit’s study confirmed that every 20% increase in tree coverage ratio caused a decrease in air temperature, with a maximum reduction of 2.7 °C on average [20]. As indicated by Zheng’s study, the greater a plant’s leaf area density, the stronger the attenuation of solar radiation [21]. Shashua-Bar’s research supportively showed that the average air temperature can be reduced by 2.5 °C in a space with a high green coverage ratio in summer (sufficient greening by trees and grassland combined) [22]. Bradley’s study found that improving green coverage is helpful for stabilizing the temperature of streets [23]. Steven’s research showed that the effect of vegetation on wind speed is subject to the differences in the leaves of deciduous trees [24]. For the areas with lower construction density in summer, based on Yuan’s research, the wind speed can drop from 0.26 m/s to 0.13 m/s as the green coverage ratio is elevated to 40% [25].

In terms of the courtyard microclimate, Guedouh found that the spatial geometry of courtyards remains the crucial decision in the optimization between thermal environment and luminous environment [26]. Rodríguez concluded that courtyard configuration has an evident impact on the improvement of human thermal comfort conditions at a pedestrian level, and the orientations and aspect ratios of courtyards influence the thermal environment directly [27]. Nasrollahi investigated the microclimate of courtyards in Cuba, with results that suggest using configurations of a high H/W (Height/ Width) rate and southward orientation to obtain better shading during summer, as well as allowing the solar radiation in while regulating the wind speed in winter [28]. Berkovic investigated the thermal environment and thermal comfort of courtyards under various inner corridor forms, greening states, and horizontal shading, and pointed out that enhancing horizontal shading is a means to improve the thermal environment and comfort of a courtyard [29]. After a survey on the thermal comfort of a courtyard in Israel, Shashua-Bar concluded that the most economical way to enhance the thermal environment is to increase the number of trees, as grass requires a relatively higher amount of irrigation but does little to improve thermal comfort [30]. Watanabe’s research suggests that the forms of courtyard enclosures, sky view factor, orientation, and aspect ratio all have a direct effect on indoor light availability, wind conditions, and construction energy consumption [31]. Muhaisen indicated that the sky view factor of a courtyard plays a significant role in the availability of solar radiation to the courtyard, which furthermore affects the cold and heat loads of buildings in winter and summer [32]. The SVF (sky view factor) is defined as “the ratio of the visible sky at a point to the sky hemisphere in a space” [33]. It indicates the capability of urban space to receive solar radiation, a value that is determined by the architecture and greening [34]. Chudnovsky pointed out that SVF defines the closure degree of an outdoor space, and functions as an important element of urban heat islands [35]. Many studies have shown that, in a city, areas with lower SVF values usually have lower daytime temperatures, which is because they harvest smaller amounts of solar radiation during the daytime [36,37,38,39,40]. Chatzipoulka also showed a significant linear relationship (*R*^2^ > 0.8) between SVF and annual global irradiance in all orientations, and the strong impact of the solar altitude angle on the relation between SVF and the amount of solar radiation obtained [41]. Ahmadi found that the SVF value imposes a limited effect on outdoor air temperature in summer, while it greatly affects the T_g_ (globe temperature) [42]. Yang’s research noted that SVF also has a large influence on wind speed [43]. 

Comparing the results of various studies, distinctions have been found in the adjustment that different spatial factors render on the microclimate under different climatic and environmental conditions. Therefore, it is of great practical significance to investigate microclimate in accordance with different climatic characteristics.

Microclimate varies among different climatic regions. In severe cold regions, winter lasts for a long time and the microclimate environment is adverse, severely affecting residents’ comfort during outdoor activities and the utilization rate of urban public spaces. The absence of rational land resource management and green ecology design concepts in earlier years has brought about multiple negative consequences. In residential areas that were constructed early in the development of cities, there are still problems such as lower-per-capita green areas, lack of outdoor activity space, and poor quality of the microclimate environment. These issues negatively affect the daily activities of residents in these residential areas. It is thus urgent to study ways of improving microclimate environments in old residential areas of severe cold regions.

The open residential areas established in Harbin around the 1960s are defined as traditional residential areas in this paper. These residential areas mostly adopt a grid-like road network structure. Most streets there are branches with small scales (the road network scale is mostly 100–200 m [44]). Most of the building groups employ an enclosed layout, where the inner part forms a courtyard. The buildings are six to eight floors high, and placed at a high density. In the traditional residential areas of severe cold regions, the streets and courtyards are considered the most important outdoor places for residents to conduct daily activities. Therefore, the microclimate environment directly affects the comfort of residents while doing outdoor activities. This study selected Harbin, a typical city of an intensely cold region, as the object to investigate the microclimate environment of the streets and courtyards in the traditional residential areas under the influence of different factors, based on the urban microclimate data acquired by means of field measurements. 

As the severe cold regions in China are characterized by low annual precipitation and arid weather, the thermal comfort of outdoor crowds is deeply influenced by the average radiant temperature, air temperature, and wind speed, while it is not significantly affected by relative humidity [45,46]. Studies have proven that changes in relative humidity negatively correlate with air temperature [47]. Based on the reasons above, the differences between microclimates of public spaces in traditional residential areas in a severe cold city and the effect of spatial morphology on microclimates are explored in this study by analyzing differences in air temperature, globe temperature (indicated by the radiation heat felt by the human body in the surrounding environment), and wind speeds of streets and courtyards. The original contribution of this paper is to explore the different effects of spatial morphology on microclimates under the action of climate in severe cold regions, and to provide reference for future designs and decision-making.

## 2. Methods 

### 2.1. Study Area and Monitoring Sites

Harbin, a typical representative city of a severe cold region, was selected as the study area for this study. According to the meteorological data from 1988 to 2010, the monthly average air temperature reached its highest value of 23.2 °C in July and lowest value of −17.5 °C in January, which indicates a distinct annual temperature difference [46]. The relative humidity was lower in spring and autumn, when it does not rain or snow much. In contrast with the summer when the temperature is higher and the wind speed is lower, winter in Harbin has lower temperatures and higher wind speeds. Residents of Harbin not only have to face extremely low temperatures and fast gales in winter, but also sweltering temperatures and windless weather in summer. Therefore, the outdoor environment of Harbin is very harsh [48]. The field measurements were performed in winter (29 December 2016) and summer (26 July 2017). According to data from the No. 50953 weather station in Harbin, which belongs to the National Meteorological Information Center of China, the temperature was approximately −22 to −14 °C and the southwest wind blew at 3.9–5.1 m/s (wind speed was measured at 10 m from the ground) in winter, while in summer the temperature was 19–28 °C and the southwest wind blew at 3.4–4.6 m/s (wind speed was measured at 10 m from the ground). Therefore, the weather conditions of the measurement days are consistent with the typical meteorological characteristics of Harbin mentioned above.

**Figure 1:**
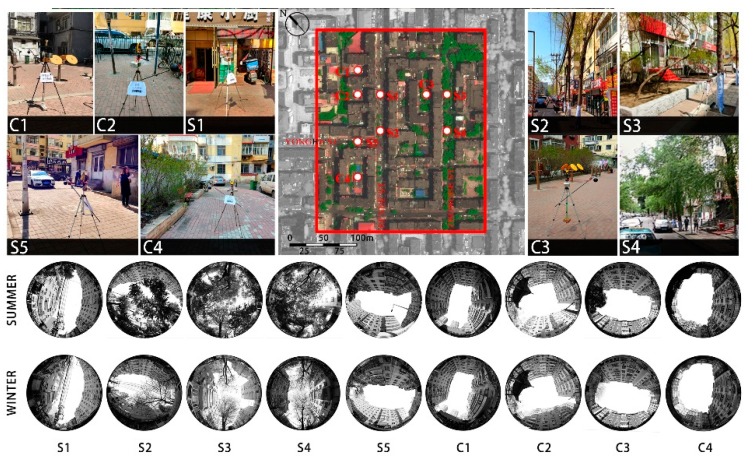
Locations of meteorological measurements and fisheye photos of each monitoring site in summer and winter.

To investigate microclimates in the public spaces (streets and courtyards) of a traditional residential area, Lujiajie block was chosen as the study area. As shown in Figure 1, five fixed monitoring sites were placed along the streets, labeled as S1, S2, S3, S4, and S5, and another four fixed monitoring sites were placed in the courtyards, marked as C1, C2, C3, and C4. These monitoring sites were distributed in pedestrian spaces and courtyards at a certain distance from the buildings, in order to avoid interference from architectural thermal radiation. Among the monitoring sites, S1 and S5 were located in non-vegetated open spaces, while the other sites were all in vegetated spaces to different extents.

### 2.2. Monitoring Instruments

A temperature collection recorder BES-01 (Institute of building energy saving technology of Harbin Institute of Technology, Harbin, China), temperature-humidity collection recorder BES-02 (Institute of building energy saving technology of Harbin Institute of Technology, Harbin, China), and meteorological station Kestrel 5500 were utilized to record globe temperature (T_g_), air temperature (T_a_), and wind speed, respectively. All instruments were calibrated and tested prior to measurements. The temperature–humidity collectors were placed in a naturally ventilated aluminum-foil hood during the measurements to prevent interference from solar radiation. The measurement devices were held by a tripod at a height of approximately 1.5 m from ground.

### 2.3. Admeasurement and Calculation of Research Elements

The urban microclimate environment mainly depends on: (1) the geometry of the built environment (primarily referring to buildings) [49]; (2) land surface characteristics and materials [50,51]; and (3) human activities [52]. With SVF as the influencing factor, this study analyzed the geometric differences of the built environment. Meanwhile, fisheye photos of each monitoring site were taken using a Cannon fisheye lens (EF 8–15mm, f/4L, USM) (Figure 1) and SVF values were computed by Rayman 1.2. Because of the influence of the vegetation on the SVF in various seasons, it is necessary to calculate the SVF at each monitoring site in winter and summer, respectively. In this paper, the land surface of the sites was made entirely of red bricks, and so the surface difference was mainly due to the green coverage ratio. Previous research has indicated that the extent of the impact of the land surface on the surrounding microclimate varies in accordance with the complexity of the urban structure [53,54]. Because the object in this study was a traditional residential area in which the block size was small, the influential scope was set at 50 m. In this study, the green coverage ratio was defined as the ratio of the sum of the green objects’ projection area to the total area of the circle, of which the center was each monitoring site and the diameter was 50 m. With reference to Krüger and Givoni’s approach of combining the field survey and Google maps, the green coverage ratio in summer was calculated by Auto CAD(version2012-education, Autodesk, San Raphael CA, USA) [54]. The spatial geometry and green coverage ratio of the monitoring sites are shown in Table 1. As the measurement time in the paper was between 13:00–17:00 on weekdays, there were not many people in the public spaces where the monitoring sites were situated. Therefore, the research did not take the impact of human activities on the microclimate environment into account.

## 3. Results

### 3.1. Influence of Orientation and the Green Coverage Ratio on the Street Microclimate

#### 3.1.1. Street Orientation

**Figure 2:**
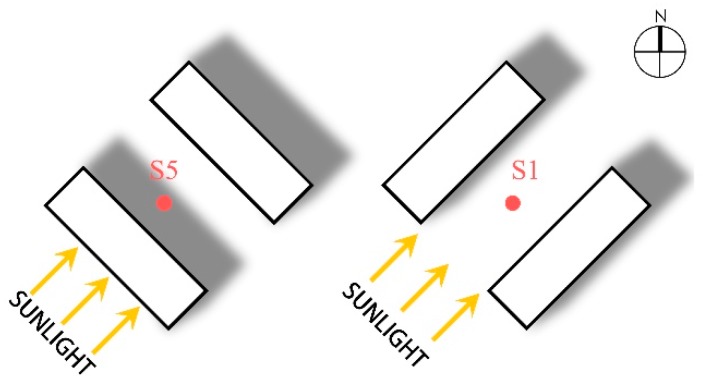
Monitoring sites of streets with different orientation.

Comparing the microclimate parameters at monitoring sites S1 and S5 in winter and summer, the influences that street orientation imposed on the microclimate were investigated. These two sites were equal in distance from the street intersection, and relatively consistent in SVF, green coverage ratio, and street aspect ratio (Table 1). Their main difference was the orientation of the street on which they were placed. S1 was situated in Renhei Street with a NE-SW orientation, while S5 was placed in Yonghe Street with a NW-SE orientation (Figure 2).

**Figure 3:**
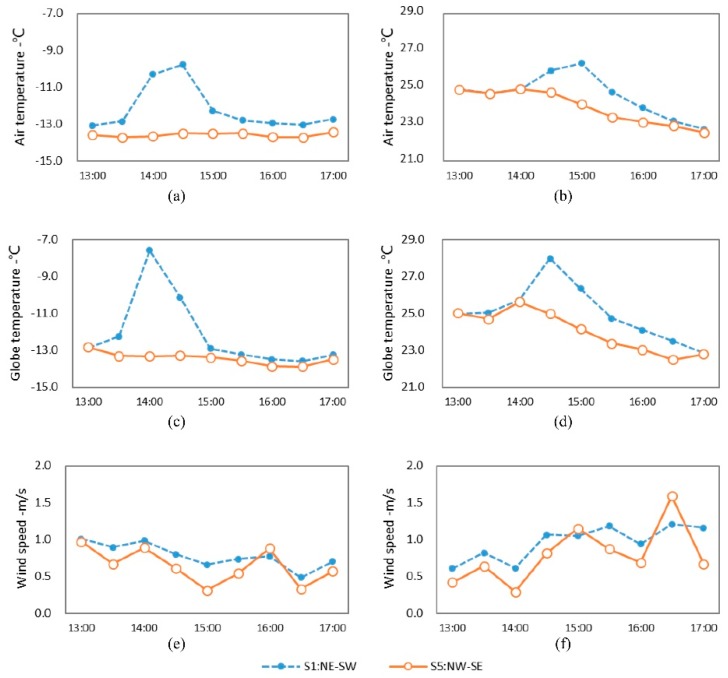
Variation of microclimate parameters of streets with different orientations. (**a**) T_a_ in winter; (**b**) T_a_ in summer; (**c**) T_g_ in winter; (**d**) T_g_ in summer; (**e**) wind speed in winter; (**f**) wind speed in summer.

Figure 3 presents the variation in microclimate parameters of streets with different orientations. In terms of the temperature in summer and winter, S5 showed lower air temperature (T_a_) and globe temperature (T_g_) than that of S1. In winter, the temperature of S5 fluctuated more mildly, with a T_a_ of −13.6 °C and T_g_ of −13.4 °C on average. On the contrary, S1 displayed a sharper undulation, where T_a_ and T_g_ were both −12.2 °C on average and respectively reached up to peaks at 14:30 and 14:00. The differences in T_a_ and T_g_ between S1 and S5 were 1.4 and 1.2 °C, respectively, while the maximum T_a_ and T_g_ differences at the same moment were 3.7 °C (14:30) and 5.7 °C (14:00), respectively. As can be seen above, the streets with a NE-SW orientation had a noticeably higher temperature than those with a NW-SE orientation. The reason for this phenomenon is the geographic location of Harbin at a higher latitude. Here, the sun’s altitude angle is relatively low and the streets in a NW-SE orientation are always situated in the shadow of buildings, which leads to the short sunshine duration (Table 1). Therefore, the temperature at S5 remained at a lower level and fluctuated to a small extent. Unlike S5, S1 was located in a NE-SW oriented street, which was able to receive a certain amount of solar radiation when the altitude angle of the sun raised to the highest point at noon, showing a longer sunshine duration than S5 (Table 1). The temperature at S1 accordingly was high, but it dropped down significantly after 15:00 (Figure 3). In summer, the average T_a_ and T_g_ of S5 were 23.9 and 24.0 °C, respectively, both achieving their highest point at 14:00. The average T_a_ and T_g_ of S1 were 24.6 and 25.0 °C, respectively, reaching a maximum value at 15:00 and 14:30, respectively. The temperature at S1 was higher than that of S5, with a difference of 0.7 °C in average T_a_ and of 1.0 °C in average T_g_, and the max differences at the same moment were 2.2 °C (15:00) and 3.0 °C (14:30), respectively. Despite this, S5 had a more volatile temperature in summer than in winter; its summer temperature and fluctuation were smaller than that of S1. Moreover, the time when the temperature peak appeared was different, as the S1 temperature peak was 30 min later than that of S5. It is thus concluded that the orientation of a street significantly influences temperature.

Regarding wind speed, S1 displayed higher values in average wind speeds in both summer and winter, compared to S5. In winter and summer, the mean wind speeds at S1 were 0.8 and 1.0 m/s, respectively, while the wind speeds at S5 were 0.6 and 0.8 m/s, respectively, with a difference of 0.2 m/s for both average wind speeds. The differences in the speed at the same moments were 0.49 m/s in summer and 0.35 m/s in winter. Those differences resulted from the dominant wind directions on the monitoring day, which were both southwest. The street in the NE-SW orientation (S1) shared the same direction as the wind, and the street scale was small, thereby inducing a narrow tube effect, which then led to a larger wind speed along the street. However, buildings obstructed the airflow, so the NW-SE oriented street (S5) appeared to have a slower wind speed and more stable wind environment.

#### 3.1.2. Green Coverage Ratio

**Figure 4:**
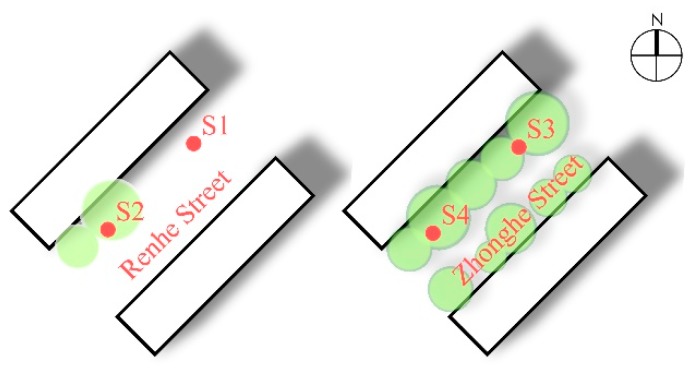
Monitoring sites of streets with different green coverage ratios.

The influence of the green coverage ratio on microclimate can be discussed by comparing the microclimate parameters of Renhe Street (S1, S2) and Zhonghe Street (S3, S4) in winter and summer. Renhe Street and Zhonghe Street are two adjacent streets in parallel, both with a NE–SW orientation and the same aspect ratio of 1.15. There are only a small number of trees in Renhe Street, while Zhonghe Street is rich in plant diversity, with dense trees and a certain number of shrubs. The greening coverage rate of monitoring sites in the two street is shown in Table 1. The microclimate parameters of each street are computed according to the average meteorological data acquired from every monitoring site in the streets (Figure 4).

**Figure 5:**
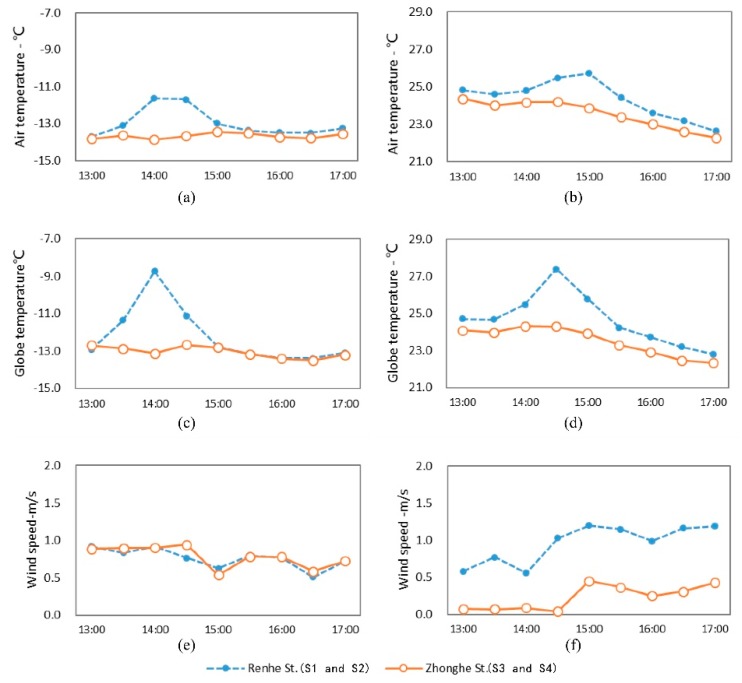
Variation in microclimate parameters of streets with different green coverage ratios. (**a**) T_a_ in winter; (**b**) T_a_ in summer; (**c**) T_g_ in winter; (**d**) T_g_ in summer; (**e**) wind speed in winter; (**f**) wind speed in summer.

Figure 5 shows the variation curves of microclimate parameters of streets with different green coverage ratios. In terms of temperature in winter and summer, the Ta and Tg of Renhe Street were both significantly higher than that of Zhonghe Street. In winter, the temperature of Zhonghe Street fluctuated slightly, with a mean Ta of −13.7 °C and a mean Tg of −13.1 °C. In contrast to Zhonghe Street, Renhe Street was more volatile in temperature. Its average Ta and Tg were −13.0 and −12.2 °C, respectively, both peaking at 14:00 (at −11.6 and −8.7 °C, respectively). The differences in average Ta and Tg between the two streets were 0.7 and 0.9 °C, and the maximum differences at the same moments were 2.2 and 4.4 °C (14:00). Although the leaves of trees fall off in winter, according to the data, the trunks shield the street from solar radiation and the vegetation on the street drops the street temperature down to a certain degree. In summer, the average Ta and Tg in Zhonghe Street were both 23.5 °C; both were relatively stable during 13:00–15:00 and then slowly fell after 15:00. The means of Ta and Tg in Renhe Street were 24.4 and 24.7 °C, respectively, and reached their highest points (25.7 and 27.4 °C) at 15:00 and 14:30, respectively. The differences between the two streets in mean Ta and Tg were 0.9 and 1.2 °C, respectively, while the max differences at the same moment were 1.9 °C (15:00) and 3.1 °C (14:30). This is attributed to the high green coverage ratio and rich vegetation composition of Zhonghe Street, where, in summer, the transpiration of leaves and their obstruction of solar radiation effectively decrease the temperature. Compared with the temperature difference in winter, both streets showed a larger difference in summer. This suggests an improved shielding effect, where arbor and shrub leaves block solar radiation. Furthermore, the plants transpire more in summer, resulting in an increased temperature difference between the two streets and a better cooling performance than that in winter.

In regards to wind speed, the differences between the two streets vary in summer and winter. In winter, Renhe Street and Zhonghe Street had similar wind speed and fluctuation, with an average wind speed of 0.8 m/s for both streets. In summer, however, the wind speed of Renhe Street was higher than that of Zhonghe Street. The average wind speed of the former was 1.0 m/s, while that of the later was 0.2 m/s, with a difference of 0.8 m/s in average wind speed. It is known that, in winter, deciduous trees provide a very weak resistance to air currents, and thus have a limited effect on reducing wind speed. In contrast, the dense leaves of trees render an obvious hindrance to wind speed, resulting in an apparent difference between the two streets. Hence, the street with a higher green coverage ratio had much lower wind speeds. This also suggests that the impact of greening on wind speed is mainly due to the attenuation of the airflow from plant leaves.

### 3.2. Influence of the Sky View Factor on the Courtyard Microclimate

**Figure 6:**
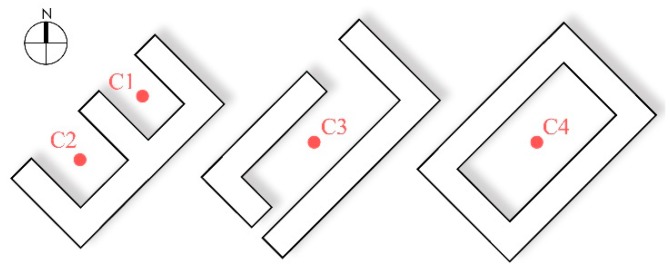
Monitoring sites of courtyards with different sky view factors.

As the green coverage ratios of each courtyard were relatively similar in the traditional residential area within the monitoring region, four distinct courtyards were selected to explore the impact of the SVF on the courtyard microclimate. C1 and C2 represented one contrast group, while C3 and C4 represented the other contrast group. Of these courtyards, C1 and C2 were located in a courtyard enclosed in three directions (there was an opening along the street on the northwest side). The SVF values at the two sites were respectively 0.22 and 0.29 in summer and 0.23 and 0.29 in winter, and the green coverage ratios were similar (0.0% and 4.7%, respectively). C3 and C4 were situated in four-side blocked courtyards, where the SVF values were respectively 0.30 and 0.37 in summer and 0.33 and 0.39 in winter, with similar green coverage rates (11.9% and 10.2%, respectively) (Figure 6).

**Figure 7:**
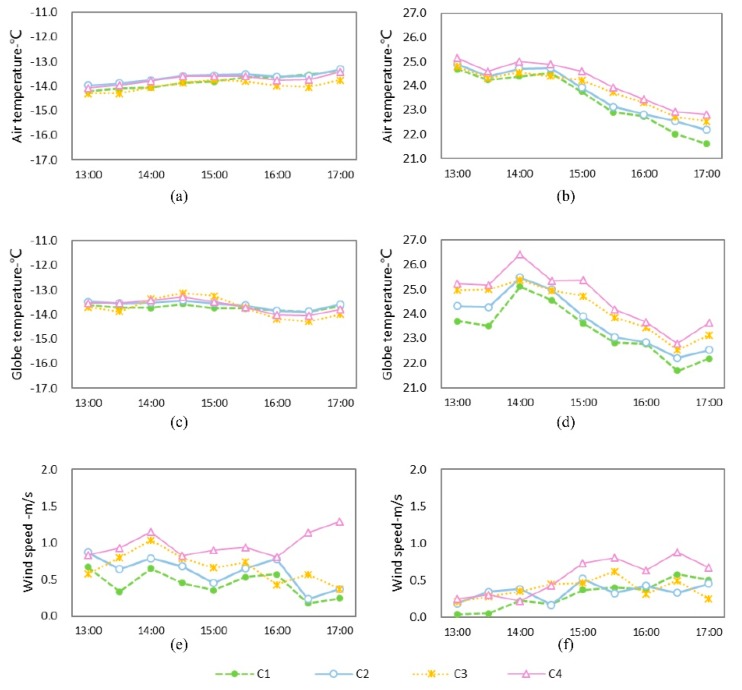
Variation curves of microclimate parameters in courtyards with different SVFs. (**a**) T_a_ in winter; (**b**) T_a_ in summer; (**c**) T_g_ in winter; (**d**) T_g_ in summer; (**e**) wind speed in winter; (**f**) wind speed in summer.

Figure 7 demonstrates variation curves of microclimate parameters in different courtyards. In regards to temperature, the four courtyards’ monitoring points showed small variations in winter and their values were close together, while in summer all points showed larger fluctuations in temperature and the differences between them were obvious. In winter, in the courtyards with a similar enclosing pattern, C1 and C2 showed closer values in mean temperature, with a difference of 0.2 °C for average T_a_ and 0.1 °C for T_g_. Moreover, the average T_a_ of C4 was higher than that of C3, with a difference of 0.3 °C, while T_g_ at the two sites had a tiny difference of 0.1 °C. The internal temperature of the courtyards was low, and the temperature difference was small at all times, as Harbin’s latitude is high and in winter the solar altitude angle is low, allowing the building to shade the monitoring point during the whole day. The sunshine duration of these for sites was 0 h (Table 1), so T_a_ and T_g_ were very similar with each other. In summer, the temperature at all four sites fluctuated greatly. T_a_ reached a maximal value at 13:00, while T_g_ reached a maximum at 14:00. Between C1 and C2, the differences in average T_a_ and T_g_ were 0.3 °C and 0.4 °C, respectively, and the difference in maximum values of T_a_ and T_g_ at the same moment were 0.6 °C (17:00) and 0.8 °C (13:30), respectively. The differences between C3 and C4 in mean T_a_ and T_g_ were 0.3 and 0.4°C, and the largest differences in the values of T_a_ and T_g_ were 0.5 °C (14:30) and 1.0 °C (14:00). It can be seen that with an increasing SVF, the courtyard had a longer sunshine duration and obtained more solar radiation (Table 1). In the case of a similar green coverage ratio, the courtyard with the higher SVF produced higher temperatures in summer. Meanwhile, the higher the summer sun’s altitude angle, the more solar radiation the courtyard could receive. Therefore, when the temperature fluctuation increased, the temperature difference between the courtyards was significantly greater during summer than in winter.

In terms of wind speed, the mean wind speed of C2 in winter was 0.2 m/s higher than that of C1, while the mean wind speed of C4 was 0.3 m/s higher than that of C3. In summer, the average wind speed of C2 was 0.1 m/s higher than that of C1, while the average wind speed of C4 was 0.2 m/s higher than that of C3. Obviously, the courtyard with a higher SVF had a higher wind speed. The reason for this is that when the greening amount is held constant, SVF can reflect the degree of spatial enclosure to a certain extent. The courtyard with a lower SVF showed a higher degree of enclosure and a more obvious impedance to the wind speed.

### 3.3. Microclimate Differences between Streets and Courtyards

**Figure 8:**
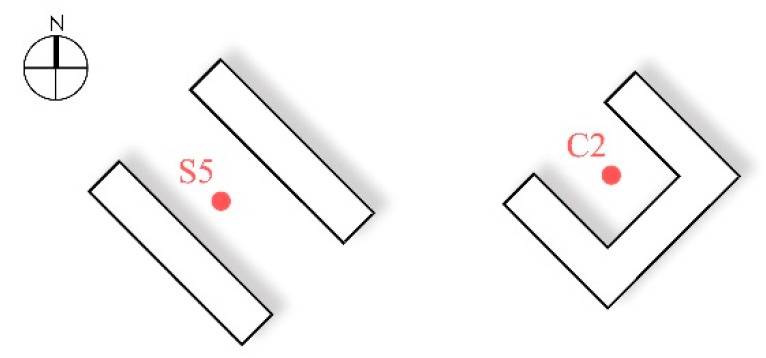
Monitoring sites of the courtyard and street with the same SVF and green coverage ratios.

By comparing the microclimate environmental parameters of S5 and C2 in winter and summer, this study investigated the microclimate differences in different public spaces. S5 was located in the street, surrounded by buildings on both sides of the street, while C2 was placed in the center of a courtyard, surrounded by buildings on three sides. Although these two monitoring sites were in public spaces with different forms, the green coverage (5.6% and 4.7%, respectively) and SVF values (0.29 and 0.29 in winter, and 0.27 and 0.29 in summer, respectively) were very close (Table 1). In addition, both monitoring sites were shaded by buildings on the northeast and southwest sides (Figure 8).

**Figure 9:**
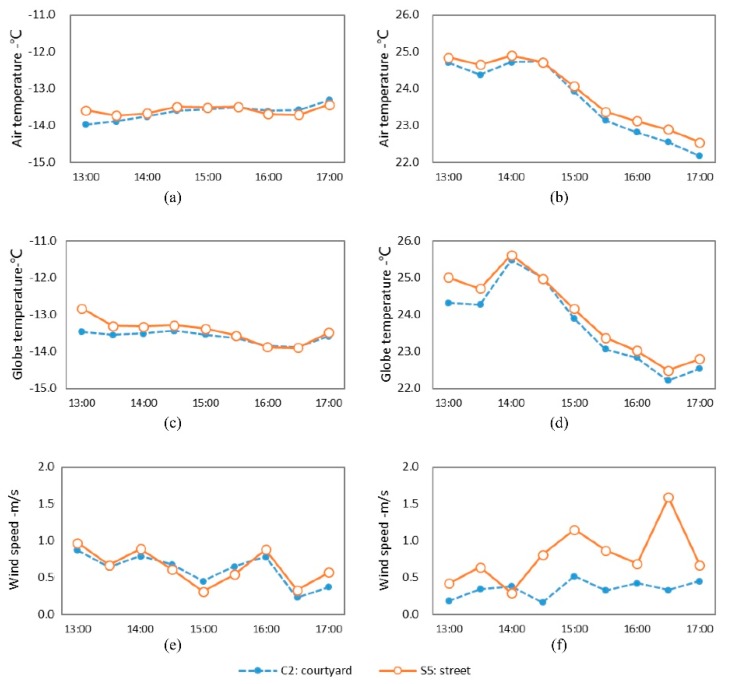
Variation curves of microclimate parameters in the street and courtyard. (**a**) T_a_ in winter; (**b**) T_a_ in summer; (**c**) T_g_ in winter; (**d**) T_g_ in summer; (**e**) wind speed in winter; (**f**) wind speed in summer.

Figure 9 represents the plot of the microclimate environmental parameters of S5 and C2. In terms of temperature, the street and courtyard generated similar temperature values in summer and winter. In winter, the temperatures in S5 and C2 fluctuated to a small extent. The mean Ta and Tg of S5 were −13.6 and −13.4 °C, respectively, while those in C2 were −13.7 and −13.6 °C, respectively. The difference in temperature was very small at only 0.1–0.2 °C. The fluctuation was more remarkable in summer. The T_a_ and T_g_ of these two monitoring sites both reached their maximum at 14:00. The temperature value of S5 was slightly higher than that of C2. The differences in the T_a_ and T_g_ were 0.2 and 0.3 °C, respectively. Therefore, even though the sites were located in different types of public spaces, their temperatures approximated each other because they had basically the same SVF values and greening conditions.

In terms of the wind speed, the wind speed of S5 in summer was obviously higher than that of C2. The mean wind speed of S5 was 0.79 m/s, while that of C2 was 0.35 m/s. The wind speed of S5 was 0.44 m/s higher than that of C2. This difference indicated that although the SVF values and the green coverage ratio were the same, the different spatial types brought about a distinction in wind speed. When parallel with the dominant wind direction, the narrow street space was more conducive to the circulation of airflow and, thus, the wind speed was faster. However, the courtyard was surrounded on three sides, leaving a hindrance to airflow, and accordingly the wind speed was lower.

## 4. Discussion

In this study, field measurements of microclimates in Harbin, a typical city of the severe cold region of China, were performed in winter and summer. It was found that street orientation, green coverage ratio, and SVF of courtyards had an influence on outdoor microclimates. Under the influence of different sunshine durations, air temperature of an NE-SW oriented street was significantly higher than that of a NW-SE oriented street, and the temperature difference between the two streets in winter was greater than that in summer. Moreover, under the influence of dominant wind direction, the wind speed of the NE-SW oriented street was higher than that of the NW-SE oriented street, a conclusion that is in accordance with previous studies [14,15,17,18]. In this study, it was found that the temperature of street with a high green coverage ratio was lower, with an air temperature difference of 0.9 °C in summer, while Shashu-Bar found that the summer air temperature in the space with a higher green coverage ratio could be reduced by 2.5 °C [22]. Due to the difference of climate background and environment, the cooling effect in this study was different from that of previous studies, but all studies prove that increasing the green coverage ratio has an obvious cooling effect. In this study, the wind speed of the street with a higher green coverage ratio was noticeably lower in summer, and the average wind speed difference was 0.8 m/s, while the wind speed of the two streets was very close in winter. Zhang’s study similarly demonstrated the reduced ability of deciduous trees to reduce wind speed in winter with a reduction of wind speed less than 0.2 m/s, whereas he noted that evergreen trees can decelerate it by more than 0.4 m/s [55]. In our study, the trees in the measurement streets were deciduous trees, providing a very weak resistance to air currents and thus a limited effect on reducing wind speed. SVF reflects the closure degree of outdoor spaces, which directly leads to differences in sunshine duration and wind speed in courtyards [37]. In this study, the courtyards with a higher SVF had a higher temperature (the temperature difference was about 0.3 °C in summer), and the wind speed of the courtyards with a higher SVF were higher (the wind speed difference was 0.1–0.3 m/s). Chatzipoulka noted that spaces with a lower SVF provide a cooler thermal environment, with a maximum difference in summer of 6 K on the hottest days [41]. Yang’s research noted the important effect that SVF has on wind speed, with each increase of 10% in SVF leading to an acceleration of 8% in wind speed [39]. Although the influence tendency of SVF on spatial microclimates was the same, the results of this study are different from the previous study, which is due to the difference in SVF value of the space.

There are still some limitations in this paper. First, there were many environmental interference factors while performing outdoor measurements, so it is difficult to absolutely eliminate the interference of environment from the measurement results. In a follow-up study, numerical simulation will be used to eliminate the influence of complex outdoor environmental factors on the research results. In addition, this study is only aimed at objective environmental differences, with a lack of discussion of thermal comfort, so in the follow-up study a questionnaire survey will be utilized to evaluate thermal comfort.

## 5. Conclusions

Using field measurements of the microclimate environments of public spaces in a traditional residential area of a severe cold region, this paper analyzed the impact of spatial geometry factors on the microclimates of streets and courtyards, and also compared the microclimate differences between different spaces. Conclusions are drawn as below:

(1) In both summer and winter, NE-SW oriented streets showed higher temperatures than NW-SE oriented streets, with temperature differences of 1.2–1.4 °C in winter and 0.7–1.0 °C in summer. NW-SE oriented streets showed smaller wind speeds in both summer and winter, and the wind speeds were more stable. The differences in average wind speed of these two streets in the two seasons were both 0.2 m/s, and the maximum difference in wind speed at the same moment was 0.35–0.49 m/s.

(2) In summer and winter, the temperatures in the streets with higher green coverage ratios were obviously lower and fluctuated to a smaller extent. The temperature differences were 0.7–0.9 °C in winter and 0.9–1.2 °C in summer, with a stronger cooling effect of plants recorded in summer. Meanwhile, the wind speeds in the streets with higher green coverage ratios were remarkably lower, with a difference of 0.7 m/s in mean speed, while in winter the wind speeds of the streets could not be distinguished.

(3) The courtyard with a higher SVF had a higher temperature in summer, and the temperature difference was about 0.3 °C. In winter, however, the difference in temperature between courtyards with different SVF values was not obvious. In both seasons, the wind speed of the courtyards with higher SVF values was higher, with a difference of 0.1–0.3 m/s between the means.

(4) When the SVF values and green coverage ratios were the same, the temperature of the streets and the courtyards were very similar in both summer and winter. The difference in average temperature was less than 0.3 °C, but in summer the wind speed of the street was obviously larger than that of the courtyard, with a difference of 0.4 m/s.

The above conclusions show that street orientation, green coverage ratio, and sky view factor have different effects on the outdoor microclimates of traditional residential areas. In order to improve the quality of outdoor microclimate environments of traditional residential areas in severe cold regions of China, it is recommended to increase the number of deciduous trees planted along the streets and increase the green coverage ratio to 30%. In this way, solar radiation will be shielded during summer, thereby reducing street thermal stress, while at the same time winter solar radiation can be ensured to fully enter the street space, raising the temperature. Due to the fact that courtyards lack sunshine in winter, and it is not realistic to improve SVF through the large-scale renovation of existing buildings, it is recommended to set up event sites and rest facilities on roofs or in NE-SW oriented streets with sufficient sunshine in winter to meet the requirement of residents’ outdoor activities. Meanwhile, windproof requirements should also be taken into consideration, where winter street activity spaces should be in the form of enclosed spaces by, for example, using shrubs or temporary construction to surround event sites.

## Figures and Tables

**Table 1:** Spatial geometric parameters and greening condition of each monitoring site.

Monitoring Site	Space	Street Orientation	Green Coverage Ratio	SVF * in Summer/Winter	Sunshine Duration in Summer/Winter(13:00–17:00)	Vegetation Description
S1	Renhe St.	NE-SW	0.00%	0.26/0.26	1.5 h/1.5 h	none
S2	Renhe St.	NE-SW	13.65%	0.12/0.19	1.5 h/1.5 h	*Salix matsudana* (Deciduous/H = 12 m/W = 7 m)
S3	Zhonghe St.	NE-SW	42.06%	0.09/0.16	1.5 h/1.25 h	*Salix matsudana* (Deciduous/H = 12 m/W = 7 m) *Syringa microphylla* (Deciduous/H = 1.8 m/W = 2.4 m)
S4	Zhonghe St.	NE-SW	30.32%	0.09/0.15	1.5 h/1.25 h	*Salix matsudana* (Deciduous/H = 12 m/W = 7 m)
S5	Yonghe St.	NW-SE	5.60%	0.27/0.29	0 h/0.75 h	*Syringa microphylla* (Deciduous/H = 1.8 m/W = 2.4 m)
C1	Courtyard	/	0.00%	0.22/0.23	0 h/0.5 h	none
C2	Courtyard	/	4.70%	0.29/0.29	0 h/0.75 h	*Syringa microphylla* (Deciduous/H = 1.5 m/W = 2 m)
C3	Courtyard	/	11.87%	0.30/0.33	0 h/2 h	*Syringa microphylla* (Deciduous/H = 1.5 m/W = 2 m)
C4	Courtyard	/	10.23%	0.37/0.39	0 h/2.5 h	*Syringa microphylla* (Deciduous/H = 1.5 m/W = 2 m)

* means sky view factor.
